# ESR1, HK3 and BRSK1 gene variants are associated with both age at natural menopause and premature ovarian failure

**DOI:** 10.1186/1750-1172-7-5

**Published:** 2012-01-17

**Authors:** Yingying Qin, Mei Sun, Li You, Deying Wei, Jielin Sun, Xiaoyan Liang, Bo Zhang, Hong Jiang, Jianfeng Xu, Zi-Jiang Chen

**Affiliations:** 1 Center for Reproductive Medicine, Provincial Hospital Affiliated to Shandong University, National Research Center for Assisted Reproductive Technology and Reproductive Genetics, The Key laboratory for Reproductive Endocrinology of Ministry of Education, Shandong Provincial Key Laboratory of Reproductive Medicine, Jinan, China; 2 Department of Obstetrics and Gynecology, Provincial Hospital Affiliated to Shandong University, Jinan, China; 3 Center for Cancer Genomics, Wake Forest University School of Medicine, Winston-Salem, North Carolina, USA; 4 Center for Reproductive Medicine, the Sixth Affiliated Hospital of Sun Yat-Sen University, Guangzhou, China; 5 Center for Reproductive Medicine, Maternal and Child Health Hospital in Guangxi, Guangxi, China; 6 Center for Reproductive Medicine, 105th Hospital of PLA, Anhui, China; 7 Fudan-VARI Center for Genetic Epidemiology, Fudan University, Shanghai, China

**Keywords:** Premature ovarian failure (POF), Primary ovarian insufficiency (POI), Age at natural menopause (AANM), HK3, ESR1, BRSK1, Premature menopause, Hypergonadotropic hypogonadism

## Abstract

**Background:**

Premature ovarian failure (POF) is a complex and heterogeneous disorder that is influenced by multiple genetic components. Numerous candidate gene studies designed to identify POF susceptibility loci have been published, but most positive findings have not been confirmed in follow up studies. We sought to determine if sequence variants previously associated with age at natural menopause (AANM) or early menopause (EM) contribute as well to genetic susceptibility to POF.

**Methods:**

Our study was performed on 371 unrelated idiopathic women with POF and 800 women controls, all Chinese Han. Thirty six SNPs from previous genome-wide association studies (GWAS) responsible for AANM or EM and 3 additional SNPs in ESR1, and 2 additional SNPs in PTHB1 were tested using the Sequenom MassARRAY iPLEX platform for genotyping.

**Results:**

Three SNPs - rs2278493 in HK3, rs2234693 in ESR1 and rs12611091 in BRSK1 - showed nominally significant association with POF. Thus, a plausible relationship could exist between ESR1, BRSK1, HK3 and POF.

**Conclusions:**

This largest association study undertaken to determine correlation between POF and AANM/EM revealed three significant SNPs (rs2278493, rs2234693, and rs12611091). All are associated with not only AAWM and EM but also POF. Insights into shared genetic susceptibility between POF and AANM/EM will provide novel entry points for unraveling genetic mechanism involved in ovarian reserve and oocyte aging processes.

## Background

Natural menopause is one of the most important physiological events, the cessation of ovarian function and the end of the reproductive lifespan. Twin and family studies indicate high heritability for age at natural menopause (AANM) (49-87%) [1]. The AANM in Chinese women is approximately 49 years [2]. Early onset of menopause increases risk for many postmenopausal health complications. Menopause occurring between 40 and 44 years has an incidence of 5% and is termed early menopause (EM) [3]. Premature ovarian failure (POF), also termed as primary ovarian insufficiency (POI), is defined as ovarian failure before the age 40 years [4,5].

POF is characterized by secondary amenorrhea, hypoestrogenism and elevated gonadotropin serum levels (FSH > 40 IU/L). Approximately 1% of the population has POF before age 40 years; only 0.1% or less are affected prior to age 30. Although most cases of POF occur sporadically, approximately 10 - 15% of cases have an affected first-degree relative [6]. Based on either known roles in folliculogenesis or phenotypes of murine knockout models, many candidate genes have been interrogated to determine their role in POF. However, except for FSHR in Finnish women none explains more than 1-2% of POF in any given ethnic group [7]. Genes whose perturbations cause POF have nonetheless been identified in single patients or families, small patient groups or selected ethnic groups. This may reflect genetic heterogeneity, ethnic-specific genetic variation or failure to interrogate sufficient numbers of the candidate genes that could potentially account for POF.

Via genome-wide association studies (GWASs), it is possible to identify common genetic variants contributing to susceptibility to genetically complex or polygenic diseases. Despite the attractiveness of GWAS for POF, genome searches in a sufficiently large number of patients are practically absent [8]. Kang H.J. et al. showed association of two SNPs (rs3884597 and rs6944723) in the PTH-responsive B1 gene (PTHB1) in a small discovery set of 24 women and 24 controls, followed by confirmation in 101 cases and 87 controls [9]. The other GWAS showed association with ADAMTS19 in the discovery set of 99 Dutch women and 181 controls, but not in the replication set (60 POF cases and 90 controls) [10]. That 8% of the relatives of women with POF in Chinese suffer from either early menopause or POF (Table 1) suggests an interrelated genetic mechanism between POF and early menopause. Indeed, Tibiletti et al. proposed that POF and EM patients share similar genetic features and postulated that these conditions may be a variable expression of the same genetic disease [11]. Additionally, FMR1 is a single gene cause of POF, and it is clinically indicated to test for premutation in this gene. Genes like EIF2B5 and FMR1 that are known to be involved in POF have been reported to be associated also with AANM [12-14]. Few studies have explored the shared genetic associations among POF, AANM and EM. On the basis of four independent GWAS, 36 SNPs have been associated with AANM. These are located on chromosomes 2, 3, 5, 6, 7, 8, 9, 11, 12, 13, 15, 18, 19 and 20, respectively, with robust statistical evidence. Of these, 2 SNPs were related to EM [1,15,16]. Besides these 36 SNPs, we also assessed correlation between POF and three additional SNPs in the estrogen receptor 1 (ESR1) gene, given the key role of ER-α in folliculogenesis. ESR1 has also been considered a promising candidate gene for POF, with rs2234693, rs9340799 and rs1569788 of ESR1 reported to be associated with idiopathic POF [17-19]. ESR1 also has been one of the most popular candidates related with AANM in previous association studies [20,21].

**Table 1:** Clinical features of cases and controls, which are not significantly different (p > 0.05).

Characteristic	
Cases	
Number	371
FSH concentration (IU/L)^a^	73.88 ± 30.23
Familial clustering^b^	32
Mother affected	27
Menopause < 40 yrs	13
40 yrs < Menopause < 45 yrs	14
Siblings affected	5
Menopause < 40 yrs	5
Age at diagnosis (yrs)^a^	29.79 ± 5.31
Amenorrhea Secondary	228
Age at menarche (yrs)^a^	14.82 ± 2.11
Age at onset of menstrual dysfunction (yrs)^a^	23.85 ± 6.78
Age at amenorrhea (yrs)^a^	24.87 ± 5.88
Controls	
Number	800
Age(yrs)^a ^at sample	38.77 ± 11.58
Age at menarche(yrs)^a^	14.75 ± 1.45

^a ^± SD

^b ^Defined as two or more first-or second-degree female family members with either POF or early menopause (less than 45 years old), including the index POF patient.

Overall, we sought to determine the contribution to POF of the 36 SNPs from AANM GWAS, the 3 SNPs in the ESR1 gene and the 2 SNPs in the PTHB1 gene. Our association study involved 371 women with POF and 800 controls, all Chinese Han.

## Methods

### Participants and phenotypes

Our study was performed on 371 unrelated idiopathic women with POF and 800 women controls, all Chinese Han, collectively gathered from a number of high volume regional referral centers in China. Samples were collected from 18 provinces during 2002-2010. The age of secondary amenorrhea in our sample was young (mean age 23.85 ± 6.78 years) (Table 1). 8% (32/400) of cases had a positive family history, i.e., at least two first-or second-degree female family members with POF or early menopause including the index POF patient. Most of the affected relatives (27/32; thirteen < 40 yrs and fourteen < 45 yrs) were mothers of the index patient; the remaining five were sisters. Inclusion criteria for POF consisted of cessation of menstrual cycles before 40 years of age, with at least two serum follicle stimulating hormone (FSH) concentrations exceeding 40 IU/L. Serum FSH level was determined by the electrochemiluminescent immunoassay in the analyzer cobas e 601 (Roche, Switzerland). The analytical sensitivity of basal FSH was < 0.10 mIU/ml. All samples were run in the same assay. Early menopause is defined as menopause that occurs between ages 40 and 44. Women with known chromosomal abnormalities, previous ovarian surgery, chemotherapy, or radiotherapy were excluded. Controls attended the clinic because of tubal factor infertility. They were known to be menstruating regularly and confirmed by hormone assays (< 40 mIU/L) and ultrasound imaging to be free of POF-related and early menopause- related phenotypes. Informed consent was obtained from all subjects. The study was approved by the Institutional Review Board of Reproductive Medicine of Shandong University.

### Genotyping and statistical analysis

SNPs were chosen for genotyping on the basis of published findings in association studies. A total of 41 SNPs are located in LHCGR, PPARG, SRD5A1, HK3, RAP80, SYCP2L, TNF, ESR1, IGF2R, PTHB1, NBN, TGFBR1, FSHB, PGR, ANKK1, IGF1, CYP19A1, POLG, SMAD7, BRSK1, TMEM150B, MCM8 or intergenic region (Table 2). We used the Sequenom MassARRAY iPLEX platform for genotyping. The Sequenom protocol involves a multiplex PCR reaction prior to a single-base primer extension reaction. The individual SNPs are identified by using matrix-assisted laser desorption/ionization time- of- fight mass spectrometry. SNPs were excluded if the minor allele frequency was < 1%. Ultimately, 36 SNPs were genotyped in the case control cohort. Allelic association analysis was conducted using SHEsis [22]. The genotype distributions for all SNPs were tested for Hardy-Weinberg equilibrium (HWE) using the Fisher's exact test. All SNPs were in Hardy- Weinberg equilibrium. The allelic odds ratio (OR) and 95% confidence interval (CI) were estimated assuming a multiplicative model. P-values were two-tailed. An alpha of 0.05 was used to claim statistical significance.

**Table 2:** Loci selected for association analysis in POF and controls.

All samples (371 cases and 800 controls)											Gene information	Phenotype related
CHR	BP	SNP	F_MISS	HWE_P	A1	F_A	F_U	A2	P	OR		
3	12423994	rs4135280	0.008333	0.2789	C	0.2861	0.2738	T	0.5321	1.063	PPARG	AANM
5	6692179	rs494958	0.009167	0.3597	T	0.2263	0.2237	A	0.8881	1.015	SRD5A1	AANM
5	176247040	rs2278493	0.008333	0.08305	A	0.3451	0.3881	G	0.0432	0.8309	HK3	AANM
5	176255904	rs691141	0.0175	0.682	T	0.4747	0.4608	C	0.5302	1.057	HK3	AANM
5	176290671	rs7718874	0.01083	0.4156	T	0.4696	0.4765	C	0.7525	0.9726	RAP80	AANM
5	176311180	rs365132	0.0175	0.4133	C	0.4669	0.4744	A	0.7343	0.9704	RAP80	AANM
5	176367046	rs402511	0.01833	0.293	C	0.4707	0.4769	T	0.7792	0.9755	RAP80	AANM
5	176436169	rs244715	0.01083	0.3229	A	0.4842	0.4703	G	0.5254	1.058	intergenic	EM
6	11005474	rs2153157	0.0275	0.5569	G	0.3484	0.335	A	0.5233	1.061	SYCP2L	AANM
6	31648292	rs909253	0.01333	0.0382	C	0.4134	0.3829	T	0.1564	1.136	TNF	AANM
6	152205028	rs2234693	0.01	0.7176	C	0.3655	0.4217	T	0.009057	0.79	ESR1	POF/AANM
6	152205074	rs9340799	0.006667	0.7148	G	0.189	0.2016	A	0.4703	0.9228	ESR1	POF/AANM
6	152370309	rs1569788	0.008333	0.7268	T	0.453	0.474	C	0.338	0.9191	ESR1	POF/AANM
6	160419555	rs2297362	0.008333	0.9062	C	0.4301	0.4383	T	0.7047	0.9669	IGF2R	AANM
6	160423646	rs9457827	0.01417	0.9059	T	0.4312	0.4379	C	0.7602	0.9732	IGF2R	AANM
7	33351787	rs3884597	0.009167	0.222	T	0.467	0.4784	G	0.6047	0.9554	PTHB1	POF
7	33390485	rs6944723	0.01083	1	A	0.4459	0.4183	T	0.2051	1.119	PTHB1	POF
8	91019056	rs2697679	0.009167	0.5546	G	0.4195	0.437	T	0.4217	0.931	NBN	AANM
8	91082415	rs7011299	0.015	0.5509	G	0.4237	0.4202	C	0.8727	1.014	NBN	AANM
9	100955986	rs1590	0.0075	0.1803	G	0.4581	0.466	T	0.7184	0.9687	TGFBR1	AANM
11	30196754	rs11031010	0.0125	0.4482	A	0.04319	0.04047	C	0.7559	1.07	FSHB	AANM
11	112786283	rs6279	0.0075	0.05507	C	0.4619	0.4747	G	0.5609	0.9501	ANKK1	AANM
13	111017433	rs1361542	0.005833	0.396	A	0.03665	0.03391	G	0.7332	1.084	intergenic	AANM
13	111017854	rs1756091	0.006667	0.3965	A	0.03655	0.03461	C	0.8101	1.058	intergenic	AANM
13	111019298	rs7333181	0.0075	0.3965	A	0.03655	0.03465	G	0.8143	1.057	intergenic	AANM
13	111019632	rs1163623	0.003333	0.3965	C	0.03627	0.03457	T	0.8326	1.051	intergenic	AANM
15	49335997	rs11856927	0.005833	0.6833	C	0.4532	0.4641	A	0.6187	0.9572	CYP19A1	AANM
15	87670333	rs2351002	0.01083	0.5205	G	0.3521	0.3398	C	0.5542	1.056	POLG	AANM
18	44721958	rs4939833	0.0075	0.1067	G	0.03403	0.04203	A	0.3495	0.803	SMAD7	AANM
19	60492141	rs12611091	0.005833	0.8633	T	0.2422	0.2001	C	0.01892	1.278	BRSK1	AANM
19	60506693	rs1551562	0.01917	0.161	G	0.06016	0.06974	A	0.3858	0.8539	BRSK1	AANM
19	60511657	rs1172822	0.008333	0.384	T	0.0953	0.09046	C	0.7026	1.059	BRSK1	AANM
19	60516144	rs7246479	0.0075	0.4537	C	0.1929	0.1895	A	0.8435	1.022	BRSK1	AANM
19	60523000	rs2384687	0.006667	0.4769	C	0.08877	0.09023	T	0.9072	0.9822	TMEM150B	AANM
19	60525476	rs11668344	0.005833	0.4762	C	0.08724	0.08962	T	0.8488	0.9709	TMEM150B	AANM
19	60525566	rs897798	0.0075	0.6029	C	0.08924	0.09383	T	0.7183	0.9463	TMEM150B	AANM
11	30203352	rs621686	0.01417	1	G	0.002653	0.00062	A	0.1956	4.285	FSHB	AANM
11	30218544	rs7951733	0.003333	1	G	0	0.000617	A	0.4899	0	FSHB	AANM
11	100477546	rs619487	0.003333	1	C	0.003886	0.006173	A	0.4769	0.6281	PGR	AANM
12	101388555	rs1019731	0.0075	1	T	0.002611	0.003094	G	0.8388	0.8435	IGF1	AANM
20	5896227	rs16991615	0.01167	1	A	0.001316	0.001241	G	0.9617	1.061	MCM8	EM

CRH: Chromosome; SNP: Single-nucleotide polymorphism; HWE_P: Hardy-Weinberg principle; F_A, F_U indicate the allele frequency in cases and controls, respectively

## Results

We analyzed 41 SNPs for association with POF in our collection of 400 cases and 800 controls. The analysis was nominally significant for three SNPs: rs2278493 in HK3, rs2234693 in ESR1 and rs12611091 in BRSK1, respectively. Results are summarized in Table 2. These findings demonstrate that certain SNPs associated with AANM also may contribute to POF, but need not necessarily play a major role in POF. No significant association with POF was observed for the remaining 38 SNPs.

## Discussion

We conducted the largest association study reported to date in a POF cohort in order to test the contribution to POF of 36 SNPs, specifically known to be associated with AANM and EM. We also investigated the associations of 3 ESR1 SNPs and 2 PTHB1 SNPs in POF. We found contribution to POF for SNPs in ESR1, BRSK1 and HK3, 2 of these genes not previously considered as candidates for POF.

Estrogen regulates cyclic gonadotropin release at the hypothalamus- hypophysis- ovarian (HPO) axis by the estrogen receptor α (ER-α), which is encoded by ESR1 and enhances follicular development by the ER-β encoded by ESR2. Female ER-α knockout mice show anovulation and complete infertility. Rs2234693 lies in an intron of the ESR1gene and has been reported to be related to POF and the onset of natural menopause in Korean and Dutch [17-19], consistent with our results. Because this SNP does not change any amino acid in the ERS1 protein, it is presumed to be in linkage disequilibrium with a regulatory sequence(s) that could affect gene expression or function and consequently result in an alteration in estrogenic biological activity.

Rs12611091 is located in the intron region of BRSK1 (BR serine/threonine kinase 1), a gene that codes for an AMP-activated protein kinase (AMPK)-related kinase [23]. BRSK1 is highly expressed in human brain and moderately expressed in mammalian ovaries [24]. Mutation or overexpression of the BRSK1 gene deleteriously affects vesicle transport and release at the axon terminals [25]. BRSK1 might affect the secretion of gonadotropin- releasing hormone (GnRH) from the hypothalamus through this mechanism. Additionally, maternal embryonic leucine zipper kinase (MELK), which is highly expressed in spermatogonia and oocytes, is one of the downstream targets of BRSK1 [26,27]. Plausible involvement in ovarian aging is therefore suggested for BRSK1.

Hexokinase 3 (white cell) (HK3), one of the four hexokinase family members, is involved in carbohydrate metabolism through phosphorylating glucose to produce glucose-6-phosphate [28]. HK3 is present in uterus, placenta, lung and adipose according to GeneAtlas [24]. Literature analysis for HK3 did not indicate an immediate functional explanation for the observed association. To elucidate the precise molecular mechanism, extensive sequence analysis and functional studies of the HK3gene variants are needed.

Idiopathic POF and EM differ in age of menopause onset and have long been considered to represent variable expression of the same genetic disease [11]. It is highly plausible that certain genes might contribute to both. That is, similar underlying genetic predisposition exists, possibly with different environmental factors or modifiers triggering disease progression and, hence, specific age of onset. Another possibility is that certain genes in pathways ordinarily predisposing only to EM interact or converge to cause even earlier age of onset, i.e, below age 40 and thus lead to clinical POF.

## Conclusions

In summary, our data demonstrate that genetic susceptibility for both AANM/EM and POF is shared by the ESR1, HK3 and BRSK1 genes. This became evident by our showing that three SNPs previously related to AANM were associated with POF. At these newly identified loci fine mapping or sequencing might lead to identification of causal variants, and thus expand our knowledge of the underlying mechanism of POF. Insights into shared genetic susceptibility between POF and AANM/EM will further provide novel entry points for unraveling etiology involved in ovarian reserve and aging process. Identifying genetic causation may further be of diagnostic value to other family members, especially female offspring. If a younger relative has the same genetic variant, timeline for achieving pregnancy might be advanced and early child-bearing encouraged.

## List of abbreviations

AANM: age at natural menopause; EM: early menopause; POF: premature ovarian failure; POI: primary ovarian insufficiency; GWAS: genome-wide association study; PTHB1: PTH-responsive B1 gene; ESR1: estrogen receptor 1; FSH: follicle stimulating hormone; SNP: single nucleotide polymorphism; HPO: hypothalamus- hypophysis- ovarian; ER-α:estrogen receptor α; AMPK: AMP-activated protein kinase; MELK: maternal embryonic leucine zipper kinase; BRSK1: BR serine/threonine kinase 1; GnRH: gonadotropin- releasing hormone; HK3: Hexokinase 3 (white cell).

## Competing interests

The authors declare that they have no competing interests.

## Authors' contributions

ZJC critically revised the manuscript, contributed to the study design, analysis, and supervised the research. YYQ drafted and critically revised the manuscript, performed the statistical analyses, and contributed to the interpretation of the data. MS, DYW, XYL, BZ and HJ coordinated clinic diagnosis, the blood collection and storage of cases and controls. YL and JLS performed genotyping and statistical analyses. JFX contributed to the bioinformatics and the interpretation of the data. All authors read and approved the final manuscript.
